# Overview of dietary intake assessment methods and dietary outcomes in Roma population: a scoping review

**DOI:** 10.1038/s41430-025-01677-z

**Published:** 2026-01-31

**Authors:** Anna Kiss, Orsolya Tompa, Sándor Soós, Zoltán Lakner, Ágoston Temesi, Brigitta Unger-Plasek, Laura Pfeiffer

**Affiliations:** 1 Department of Science Policy and Scientometrics, Library and Information Centre of the Hungarian Academy of Sciences, Budapest, Hungary; 2 Pro-Sharp Research and Innovation Centre, Budapest, Hungary; 3 Faculty of Education and Psychology, ELTE Eötvös Loránd University, Budapest, Hungary; 4 Institute of Agricultural and Food Economics, Hungarian University of Agriculture and Life Sciences, Budapest, Hungary; 5 Tashkent State Agrarian University, Tashkent, Uzbekistan

**Keywords:** Translational research, Preclinical research

## Abstract

The Roma minority is one of Europe’s most vulnerable minorities in terms of health status including nutrition-related diseases. A detailed and robust exploration of the dietary behaviors of the Roma population is essential for developing targeted nutrition interventions. This scoping review aims to identify and evaluate the dietary assessment methods used for measuring dietary intake and food consumption among the Roma population. Studies were identified through PubMed, Web of Science, Scopus, and grey literature. Studies written in English that focused on the Roma minority and assessed dietary intake and food consumption were eligible. A qualitative approach was used to summarize the results. Thirteen original studies were reviewed, primarily conducted in Eastern Europe. Nine out of thirteen studies used cross-sectional study designs and quantitative research approaches. The most commonly applied nutritional assessment methods were food frequency questionnaire, 24-h dietary recall, and brief dietary habits questionnaire. Quantitative dietary outcomes were measured through dietary intake, food consumption patterns, and dietary quality, while qualitative dietary outcomes were identified as moderate overconsumption, irregular eating, or the symbolic use of food to denote social status. There was a lack of validity and adaptation of dietary assessment tools for the Roma population. The reviewed studies often did not employ advanced nutritional analysis methods common in the general, non-Roma population. There is a need to develop specific dietary assessment methods for the Roma population. To obtain more reliable results, combining subjective dietary assessment methods with a qualitative, participatory approach may be suitable for this population.

## Introduction

Ethnic minority groups, including Roma communities across Europe, have long experienced nutritional vulnerability and distinct disease patterns compared to the general population, reflecting ongoing socioeconomic health disparities rooted in their ethnic backgrounds [1, 2]. They often experience higher disease burdens, lower life expectancy, and educational attainment, regardless of their country of residence [3, 4]. The Roma population represents the largest ethnic minority in Europe; the World Health Organization (WHO) estimates their number ~12–15 million in the European Region, out of which 6 million live in the European Union. Most of them reside in the Central and Eastern European countries of the EU [5].

The Roma population is distinct from the non-Roma population in terms of their cultural and social characteristics, language, appearance, traditions, and unique cultural identity. As a result of social integration, their traditional way of life has changed, but characteristics of lifestyle and eating have remained [6].

The dietary quality of the Roma population is greatly influenced by their eating tradition controlled by strict rules. Their rules include, for example, the prohibition of eating food prepared by non-Roma people [6, 7]. This increases the costs of producing certain foods and impose special requirements on their preparation and storage [8]. All these factors lead to a different dietary behavior of the Roma population compared to the non-Roma population.

Certain ethnic minority groups living in Europe have a high prevalence of preventable non-communicable diseases due to the adoption of less healthy, ultraprocessed diets [9, 10] among which is the Roma minority. Evidence shows that Roma communities suffer from chronic diseases (e.g., depression) and chronic NCDs (e.g. cardiovascular diseases,) to a greater extent compared to the majority population [11–15].

There has been growing attention on health status, dietary patterns, and the influence of acculturation on diet among minority populations. Although some studies examine the prevalence of NCDs among the Roma population and highlight inadequate nutrition as a risk factor, few specifically focus on their nutritional intake and food consumption. A few comprehensive studies have been conducted on the health status of the Roma by esteemed organizations such as the European Commission, the United Nations Development Programme (UNDP), and the World Bank across 17 European countries [16–19] but they do not include detailed data on the dietary habits of the Roma. A detailed description of the food consumption, dietary pattern, and nutritional status of the Roma population can be found in national studies, but a systematic review of these researches has not yet been carried out.

Different eating and food preparation habits originating in their culture influence the dietary pattern of the Roma population. In addition to food culture, beliefs about food, food availability, gender, age, income, level of education, and acculturation are all influential factors in dietary behavior and food choices [9]. To design and implement nutrition-related interventions and develop strategies for addressing inequalities in health status in the Roma population, the detailed exploration and understanding of the dietary habits and nutrition of the Roma population are essential. Holdsworth et al. emphasize the need for sensitive, innovative dietary behavior interventions tailored to the specific needs of ethnic minority populations in Europe [9].

During the assessment of ethnic minorities’ nutrition, several challenges were identified in the literature: the contribution of ethnic foods to dietary intake, assessing individual consumption from a shared serving dish/pot, the accurate estimation of portion sizes, and the lack of specific food composition databases [20]. To obtain reliable and valid data on the dietary habits and nutrient intake of the Roma population, culturally appropriate dietary assessment techniques are required.

To this day, according to our literature search, no systematic or scoping review specifically examines the dietary outcomes and dietary assessment methods among the Roma population. Therefore, this paper aims to close this gap. The primary purpose of the study was to identify and evaluate the dietary assessment methods used for measuring dietary intake and food consumption among the Roma population.

With this study, we aim to address the following research questions:

RQ1 What dietary assessment methods were used to examine the nutritional intake and food consumption of the Roma population?

RQ2 To what dietary reference the nutritional intake and food consumption of the Roma population is compared in the reviewed studies?

RQ3 Were dietary assessment methods validated and adapted for the Roma population?

RQ4 Which dietary assessment methods are most suitable for assessing the dietary habits of Roma?

## Methods

### Protocol

This scoping review was conducted following the PRISMA Extension for Scoping Reviews (PRISMA-ScR), which guided the literature search, selection process, and reporting [21] (Supplementary Table S1).

#### Identification of relevant studies

In order to identify relevant studies, the search strategy was in line with both the PICO (Population, Intervention, Comparator and Outcomes) framework and the aim of the scoping review. The main components of this tailored strategy were the following: “dietary intake”, “dietary assessment” and “Roma population”. There was no limitation on publication date, all studies published until the end of December 2023 were included in the study. For the literature searches, the most comprehensive databases were used including PubMed, Web of Science (WOS), and SCOPUS as they can be considered the main international electronic data sources of scientific publications. Due to the high number of irrelevant hits, we limited the search to the title and abstract. In addition, related references of selected publications and reviews were examined to identify potentially eligible studies. The search terms used for each database are provided in Supplementary Table S2 in the Supporting Information online.

To identify relevant studies and reports that are not included in the abovementioned databases, a manual web-based search was also carried out using Google Scholar. The web pages of Roma associations in Hungary were also searched. The European Union (EU) and international projects that examined the health and nutrition of the Roma were also reviewed on the official websites of the EU and WHO (e.g. European Economic and Social Committee).

#### Eligibility criteria

The inclusion criteria of the study were defined by using the PICO framework [22]. These criteria were the following: (1) included assessment of the nutritional intake and food consumption among the Roma population and (2) publication in English language (Table 1). Those studies also selected for inclusion that studied health status as their main purpose but also included food consumption or nutritional intake assessments. There was no limitation for the target populations in terms of age above 18 years or gender of published studies. As for the type of study design, study results were extracted from cohort, cross-sectional, retrospective, surveys, intervention, and qualitative studies. In contrast, book chapters, conference proceedings, duplicates, and non-peer-reviewed publications were excluded from the study. Further studies were excluded due to the following reasons: they have been carried out among children, non-humans, and ethnic groups not related to the Roma minority, have not recorded either nutritional intake or food consumption, and studies that reported dietary intake and food consumption but did not include the methodology they used.

**Table 1:** PICOS criteria for inclusion of studies.

Criterion	Description
Population	Roma minority populations (18 years or older), regardless of their place of residence
Intervention/interest	Any health or dietary survey or intervention, or prevention program in which a dietary assessment method was used to measure dietary intake or food consumption. Any qualitative investigations into the nutritional practices and behaviours of Roma population.
Comparison	Non-Roma population, official dietary guidelines
Outcomes	Dietary intake, eating habits, food consumption, dietary behaviour, dietary assessment method, method validation, diet-related outcome evaluated through the use of dietary assessment methods
Study design	Any study design in which a dietary assessment method is reported

#### Screening of identified papers

Two members of the research group, LP and AK assessed titles, abstracts, and full texts of articles, determined the eligibility of articles for inclusion in the study, and extracted data from eligible papers. Both researchers independently extracted distinct sets of data, and the two authors compared and discussed their results until they reached a consensus on the interpretation of the data. If the two researchers did not reach a consensus, a third researcher was involved to resolve the dissonance. Finally, all members of the research group reviewed and discussed the results of the analysis until a consensus was reached.

No quality assessment was conducted for this scoping review, as the goal was to summarize and evaluate methodological issues on the topic to inform future research, and practice, without the intention to include or exclude studies based on quality.

#### Data extraction and data analysis

Data was extracted using a standardized Excel template, which included author and year of publication, population characteristics (mean age/age range, subjects, sampling methods, setting, and outcomes), and methodological characteristics (study design, sample size, dietary assessment methods, reference time, administration of the tools, measurements of portion sizes, validation, dietary database, analysis tools). In addition, to achieve our aim of assessing the dietary outcomes of the Roma population, we extracted information on their energy and nutrient intake as well as food consumption. Data extraction was carried out with the use of the HubScience Research Intelligence Software [23].

The analysis of the studies was processed based on qualitative data synthesis. The reviewed articles were evaluated with a focus on methodology and research perspective, analyzing the dietary assessment methods employed.

## Results

**Fig. 1:**
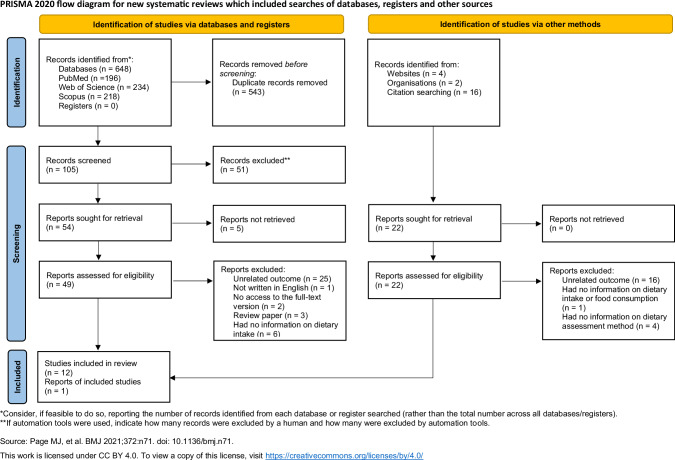
PRISMA flow diagram of the literature search process.

Overall, 648 papers were identified through database searches, and an additional 22 were retrieved via other methods, including web-based searches, and references from relevant papers. Figure 1 shows a flowchart of the selection process for the review, providing a detailed summary of the search results. After duplications were removed, the title and abstracts of 105 records were screened. Out of the 105 papers, 51 papers were excluded after the title and abstract screening because they did not meet the selection criteria. Throughout the full-text screening, 37 papers were excluded because the screening outcome did not fit with the aims of this study. The criteria-based exclusion process included the following main aspects: the record was a review paper, the full-text version was inaccessible, non-English language, and no information on nutritional intake, food consumption or eating habits. Finally, 13 studies were eligible and reviewed in this study.

### The characteristics of the reviewed studies

Most of the studies were conducted in Central and Southeastern-European countries, out of 13 studies 1 was carried out in Spain [24], 3 in Hungary [25, 26, 27], 3 in Slovakia [28, 29, 30], 4 in the Czech Republic [31, 32–34], 1 in Albania [35], and 1 in Romania [36]. As for the study design, the majority of the selected studies were cross-sectional, [25, 26, 28, 29, 31, 35, 27, 32–34] out of which 10 applied a quantitative approach, and one mixed methodology [32]. Three studies conducted a retrospective study design using existing data [24, 30, 36]. The sample sizes ranged from 150 to 797 participants in the quantitative surveys, with the exception of the two studies conducting national surveys. The number of participants was between 25 and 70 in those studies that applied either mixed or qualitative methodologies. Among the studies, only the ones conducted in Hungary (*n* = 3) [25, 26, 27] mentioned whether the sample included in the study can be considered representative. They reported that the examined Roma population can be considered representative only for the Hungarian Roma living in segregated colonies in the given region but it is not representative of the entire Roma population living in Hungary [25, 26, 27].

Among the reviewed studies, there was one Romanian study in which the diet of the Roma population at the household level was examined: for data analysis of the Household Budget Survey (HBS) (*n* = 2654) was acquired [36]. Four studies were conducted by only including Roma participants [29, 30, 32, 35], while in 9 studies there also was a control group recruited [24–26,28, 27, 31, 33, 34, 36]. They were made up of participants selected from other ethnic groups or the non-Roma population from the majority of society living in the same country.

Most studies have utilized non-probability sampling methods such as stratified two-stage cluster sampling [25, 26], random sampling [27, 33, 34], or convenience sampling [31], while in one reviewed article, the sampling method was not mentioned at all [36]. As for the place of studies, 2 studies were conducted in an urban setting [29, 35] while others had recruited participants from segregated colonies/settlements [25, 26, 28, 30] or different regions (e.g. South-Bohemian Region [32–34]. In two studies, the dietary intake and food consumption of the Roma population were examined at the national level [24, 36]. Several studies used validated dietary data recording tools [25, 26, 27, 29, 33, 34], while some did not mention whether the tools were validated or not [24, 28, 31, 35, 36, 30, 32]. As for dietary reference to compare the Roma minority’s diets with, some studies generated dietary patterns of the non-Roma population (i.e., majority population) for reference [24, 28, 31, 35, 36], some compared nutrient intakes against Recommended Dietary Intake Values, others assessed adherence to Food-Based Dietary Guidelines, or both [25, 26, 33], while the two studies using qualitative approach did not mention dietary references to compare Roma minority’s diets with [30, 32]. Specific characteristics of the reviewed articles are presented in Table 2.

**Table 2:** Summary of general characteristics of the included articles on nutrition in the Roma population.

Authors and year of publication	Country	Study design	Sample size	Time of data collection	Sampling method	Mean age/Age range	The setting of Roma population	Tools for measurement	Validation of dietary measurement tools	Reference to compare Roma dietary patterns	Outcome
Porras et al. [24]	Spain	Retrospective	1167 Roma adults and 21,007 non-Roma adult	Secondary data from previous surveys (National Health Survey 2012 and Roma Health Survey 2014)	Multistage sampling	Over 15	Nationwide	FFQ	Not indicated	Dietary patterns of different social groups	Adherence to healthy dietary patterns, guidelines
Bárdos et al. [27]	Hungary	Cross-sectional	808 adults; 393 Hungarian Roma, 415 Hungarian non-Roma	May-August of 2018	Random sampling	18–70	Segregated settlement	Anthropometric measurements, blood samples, dietary intake estimation, with repeated (2x) 24-h dietary recalls	Validated	Healthy Eating Index-2015 (HEI-2015)	Dietary quality
Llanaj et al. [25]	Hungary	Cross-sectional	797 adults; 387 Hungarian Roma, 410 Hungarian non-Roma	Not indicated	Stratified multistep sampling	20–64	Segregated colonies	Anthropometric measurements, multiple-pass 24-h dietary recall	Validated	Healthy Diet Index (HDI), Dietary inflammatory Index (DII), the EAT-Lancet and (Dietary Approach to Stop Hypertension) DASH	Adherence to healthy and sustainable dietary patterns
Llanaj et al. [26]	Hungary	Cross-sectional	797 adults; 387 Hungarian Roma, 410 Hungarian non-Roma	May-August of 2018	Stratified multistep sampling	20–64	Segregated colonies	Anthropometric measurements, multiple-pass 24-h dietary recall	Validated	Internationally accepted guidelines	Dietary intake
Ciaian et al. [36]	Romania	Retrospective	2654 Roma households, 115,978 non-Roma household and other ethnic minorities	Household Budget Survey data for the period 2004–2011	NA	45.6	Nationwide	Count of food items consumed	Not indicated	Comparison to non-Roma dietary patterns	Dietary quality, diet diversity
Olišarová et al. [32]	Czech Republic	Cross-sectional	302 Roma adults	Between 1 June 2014 and 31 March 2015	Convenience sampling	Over 18	South Bohemian region	Semi-structured interviews, in depth interviews on eating habits,anthropometric measurements	Not indicated	Not indicated	Eating habits
Sedova et al. [33]	Czech Republic	Cross-sectional	600 adults; 302 Roma, 298 non-Roma	From June 2015 to March 2016	Random proportional sample, snowball sampling	Over 18, the average age was 39.2 years for the Roma group and 44.6 years for the control group	South Bohemian region	Anthropometric measurements, 24-h dietary recall and frequency foodstuff questionnaire	Validated	Association for Nutrition (SPV) dietary recommendation	Dietary intake, eating habits
Kozubik et al. [30]	Slovakia	Ethnographic field research	More than 70 Roma adults	Summer months of 2012 and 2013	Random sampling	NA	Segregated settlement	Journal reports from 1775 and semi-structured interviews	No (semi-structured interviews)	Not indicated	Food and eating habits
Dolák et al. [34]	Czech Republic	Cross-sectional	600 adults; 302 Roma, 298 non-Roma	Not indicated	Random proportional sample	18–70 or over	South Bohemian region	Semi-structured interview on eating behaviors, 24-h dietary recall, frequency analysis of selected foodstuff, anthropometric measurements	Validated	Compared to non-Roma dietary patterns	Food consumption
Hijová et al. [28]	Slovakia	Cross-sectional	855 adults; 452 Roma, 403 Slovakian non-Roma	2011	Convenience sampling	18–55; average age of the Roma group was 34.47 (±9.16) years, the average age of non-Roma respondents was 33.47 years	Separetad settlement	Questionnaire on breakfast eating habits and consumption of different foods	Not indictaed	Compared to non-Roma dietary patterns	Eating habits
Hoxha et al. [35]	Albania	Cross-sectional	400 Roma adults	2010	Stratified two-stage cluster-sampling	36.5 (±14.2)	Urban	FFQ, anthropometric measurement	not indicated	Comparison of dietary patterns based on survey results	Dietary habits and patterns
Rambousková et al. [31]	Czech Republic	Cross-sectional	76 Roma women and 151 women from the majority population	From 2000 to 2002	Convenience sampling	Average age of the Roma women 25.4 ± 5.2, non-Roma women 26.9 ± 4.2	NA	FFQ, use of food supplements during pregnancy	Not indicated	Compared to non-Roma dietary patterns	Dietary intake
Siváková et al. [29]	Slovakia	Cross-sectional	150 Roma adults (68 males, 82 females)	Not indicated	Convenience sampling	Males: 42.1 (±13.9), females: 40.9 (±13.7)	Urban	Validated FFQ and single 24-h dietary recall	Validated	Guidelines for atherosclerosis prevention, Slovak Recommended Dietary Allowances (RDVs)	Dietary intake

### Dietary assessment methods

Dietary data collection and assessment methods as well as the results of the selected studies were reviewed – for pragmatic reasons – according to the methodological approach classified as follows:

1. Quantitative approaches
  - 24 h dietary recall surveys
  - Tools for assessing dietary quality
2. Qualitative (portions not included) or Quantitative (portions included) Food Frequency Questionnaires
3. Qualitative approaches
  - interviews
  - document analysis

In several studies, 24-h dietary recall and FFQ were both used, so these studies are mentioned in the supporting information tables multiple times in the detailed analysis of the respective methodology.

### Dietary assessment and results based on data from 24-h dietary recall

Three of the four studies that used the dietary recall reported using a single recall for the indicated reference time [29, 33, 34] while one study chose multiple recalls [26]. The studies that used multiple recalls, reported that the number of recorded days were not consecutive. One of the studies described using either food images or kitchen utensils for the description of portion sizes [26] while three studies did not mention how portion sizes were measured. Two studies identified the food composition table/database they used in their studies [26, 29]. One of them described the use of a food composition database while in other studies, the authors did not describe which database was used. Three of four studies that used a 24-h dietary recall reported that the tool was validated [26, 33, 34], and one study did not describe the validation process [29]. National or self-developed nutrition software was used to analyze the dietary data in three studies [26, 29, 33], one study did not describe the analysis tool they used [34]. Two studies reported that dietary assessments were conducted by a trained interviewer [26, 34]. Only one study focused on the adaptation of study instruments to the targeted Roma population by identifying commonly consumed ethnic foods and dishes widely used kitchen utensils or expanding food composition tables with traditional foods [26]. The main form of administration was face-to-face interviews, which were conducted with the help of trained interviewers. The outcome of the studies using 24-h dietary recall was the nutritional intake of the Roma respondents in terms of daily energy, macro-, and micronutrient intake amounts.

The results of Llanaj et al. [26] showed that the mean daily intake of fat and protein was higher than the recommended dietary allowance originating in a high proportion of animal-based protein and cholesterol intake. Total carbohydrate daily intake was lower than the recommended according to internationally accepted guidelines, however, significantly higher sugar intake was reported [26]. Dietary intakes of micro-nutrients and dietary fiber did not meet reference values [26, 29]. Specific details from studies that used 24-h dietary recall have been summarized in Supplementary Table S3 in the Supporting Information online.

### Dietary quality assessment and results based on calculated dietary quality scores

In addition to the assessment of nutritional intake and food consumption of the Roma population, 4 studies examined dietary quality with dietary quality scores as tools [24, 25, 27, 36]. Ciaian et al. [36] used an econometric approach in their study; diet diversity was measured by the number of food items consumed. The Simpson index, and by Entropy index and the data required for the analysis was acquired from Household Budget Survey data. This was the only research that examined food consumption at the household level. In the other three studies, individual-level nutrient-based dietary patterns or food intake data formed the basis of the analysis [24, 25, 27]. Complex indices were applied to assess dietary quality: Healthy Eating Index-2015 (HEI-2015), Healthy Diet Indicator, Dietary Inflammatory Index, Dietary Approaches to Stop Hypertension, EAT-Lancet [24, 25, 36]. Detailed information on studies that assessed dietary quality has been provided in Supplementary Table S4 in the Supporting Information online.

In these studies, the results on the following dietary quality scores were analyzed and compared: diet diversity gap between Roma and non-Roma respondents, adherence to healthy eating, or alignment to food-based dietary guidelines [24, 25, 27, 36]. The results of the study of Ciaian et al. [36] showed that there is a significant gap in diet diversity between Roma and non-Roma groups. Furthermore, the Roma population’s dietary quality was significantly lower according to HEI and econometric indexes too [27]. The Roma ethnicity had no significant effect on healthy nutrient-based patterns while poor adherence to selected dietary guidelines (DII, HDI, DASH, EAT-Lancet) has been shown in both populations, however, Roma ethnicity was associated with lower DII [25]. According to the results of Porras et al. [24] the Roma population have a lower adherence to the Spanish dietary guidelines compared to the non-Roma population. Their analysis showed that age is the most influential variable in food consumption, and this tendency is even more pronounced within the Romani population.

### Dietary assessment and results based on data from food frequency questionnaire

From the set of the reviewed articles, 7 studies used FFQ [28, 29, 31, 35, 32–34] for recording dietary data, out of which 5 carried out the survey in the frame of face-to-face interviews, while in only 2 studies it was mentioned that the data was collected with the help of a trained interviewer. None of the studies had information on the inclusion of ethnic foods in the FFQ, the source of the FFQ, and whether the FFQ was adapted to the Roma population. Three studies have reported the reference time of the FFQ [28, 31, 32] while another study has reported that they have used a FFQ specifically developed for the survey [31]. There were only two studies that have described the number of foods/food groups included in the FFQ [28, 32] and one study has reported the most frequently consumed foods provided by FFQ [35].

One study has applied a more complex statistical method (i.e. factor analysis) to evaluate the dietary data by which they could describe the dietary pattern of the Roma population [35].

These studies revealed the eating habits of the Roma population including food and drink consumption, frequency of meals, and evaluation of balanced healthy eating [28, 29, 31, 35, 32–34]. In terms of food consumption, the consumption of fruits, vegetables, and dairy products was significantly lower and the consumption of high-sugar drinks and side dishes such as potatoes, pasta, rice, and dumplings was higher in the Roma population compared to non-Roma respondents [28, 32, 33, 35]. According to the results of Dolák et al. [34] the eating habits of the focused Roma population were characterized by irregularities compared to the majority Czech population. Specific details from studies that used FFQ can be found in Supplementary Table S5 in the Supporting Information online.

### Dietary assessment and results based on qualitative methods

The dietary habits of the Roma population were investigated through semi-structured and in-depth interviews, as well as document analysis in two studies [30, 32]. Both studies used open-ended questions in face-to-face interviews to collect data. The questions were tailored to the purpose of the studies, but the source of the questions was not mentioned. One of these studies was based on an ethnographic field research [30]; the authors used classical sociological theory to interpret the obtained data. The other study applied the grounded theory methodology and they used MAXQDA software to analyse the textual data [32].

In both researches, main and subcategories were created to classify the diet of the focused Roma population and the following themes and topics were identified from the analyses: food storage, traditional Roma foods, motivation for foodstuff selection, preferred preparation of foodstuff, place of eating, regularity of eating, and food as a measure of wealth [30, 32]. According to the results of this research, the nutritional culture of the Roma is characterized by irregular eating and moderate overconsumption and eating as a symbol of social status in the Roma community [32]. In addition, the findings revealed that the food and eating habits of the Roma have not significantly changed over the centuries, the nutrition of the Roma population was similar to the nutrition of the low-income non-Roma population (Supplementary Table S6 in the Supporting Information online) [30].

## Discussion

**Fig. 2:**
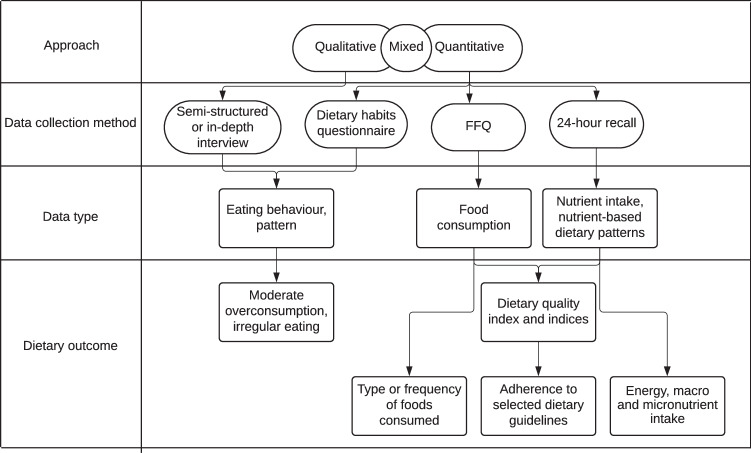
Overview of dietary assessment tools and dietary outcomes used in the reviewed studies.

In this scoping review, we reviewed the dietary assessment methods used to evaluate the dietary intake and food consumption of the Roma population. Overall, this review includes 13 studies that have applied qualitative, quantitative, or mixed methods in their dietary investigations. In the majority of the reviewed studies, retrospective direct dietary assessment methods were used to estimate food and nutrient consumption at the individual level. These methods were as follows: 24-h dietary recall, FFQ, and dietary quality scores and indices. Quantitative dietary outcomes were expressed as dietary intake, food consumption patterns, adherence to healthy eating, and dietary quality while qualitative dietary outcomes were expressed as moderate overconsumption, irregular eating, or food as a symbol of social status. Figure 2 gives an overview of dietary assessment methods and dietary outcomes based on studies included in the review.

Overall, the results of the reviewed studies suggest that dietary intake and food consumption in the Roma population show poor adherence to the selected nutritional guidelines. Our findings align with a systematic review indicating that the dietary habits of certain European ethnic groups are less healthy due to increased consumption of processed foods high in energy, fat, sugar, and salt. These foods often replace healthier components such as fruits, vegetables, nuts, and grains [10]. This could be because, similar to other ethnic minorities, the dietary habits of the Roma population are significantly influenced by the social and cultural environment, food beliefs and perceptions, and limited access to food [37].

Despite the numerous studies examining the prevalence of NCDs (e.g., obesity) in the Roma population [38, 39], very few studies have assessed the relationship between diet and non-communicable diseases in the Roma population. These studies typically use only one or two tools to evaluate dietary intake and often do not employ advanced nutritional analysis methods common in the general population. Regarding the study designs, most of the reviewed studies relied on cross-sectional study designs, which are commonly used in public health studies. This is likely because the Roma population is difficult to reach. Cross-sectional studies are suitable for assessing dietary intake at a single point in time, however, in the future, it would be beneficial to conduct, prospective, case-control, and cohort studies using advanced analysis methods to investigate the relationships between dietary intake and NCD outcomes and other nutrition-related health issues among the Roma population.

Selecting an appropriate dietary assessment method depends on the study’s purpose and these methods should be tested for accuracy and reliability in the study population. In seven studies, the food consumption and eating habits of the Roma population were analyzed using an FFQ and other dietary habits questionnaires. In these studies, the purpose of the FFQ was to assess the foods and food groups consumed, not to determine nutrient intake. Among the six studies one mentioned the reference time of the FFQ, Olisarova et al. [32] covered a week in assessing food consumption in their study. To determine the usual food consumption, it would be reasonable to take a longer period of time to map food consumption in different seasons of the year. In the examined studies, the completion of the FFQ was interviewer-administered, reducing the likelihood of incomplete responses occurring with self-administered completion. One of the strengths of the interview-based FFQ questionnaire is that it does not require reading and writing skills from the respondent, thus it is a low respondent burden for participants. One of the factors influencing the validity of the FFQ is the number of foods listed. Including ethnic and commonly consumed foods is crucial, especially in minority populations. None of the reviewed studies justified the sources or criteria for compiling the food list in the FFQ, a common shortcoming. When selecting foods, cultural significance should be considered, utilizing methods like focus groups, experimental recalls, studies on Roma dietary culture, or Roma cookbooks to compile the list.

### 24-h dietary recall method

Four studies assessed the nutrient intake or eating habits of the Roma population using the 24-h recall method. One study used the multiple-pass 24-h recall method [26], while the other three employed a single recall approach. However, a single 24-h recall is not sufficient for accurately assessing an individual’s usual nutrient intake. Using multiple 24-h dietary recalls provides a more precise measurement of average dietary variations when compared to a single recall. The interview-based 24-h recall method reduces recall bias and does not depend on the literacy of respondents. However, it requires well-trained interviewers with knowledge of the given cultural practices, ethnic foods, and local recipes. One strength of this method, in addition to dietary intake estimation, is that it provides further information about participants’ eating and food preparation habits, meal locations, and meal frequency. As a result, the 24-h recall can contribute to a better understanding of the dietary culture among the Roma population.

When using both 24-h dietary recalls and FFQs in dietary assessments, the estimation of portion sizes is a critical challenge, especially in low-resource settings where standard models and sizes are often lacking. Among the analyzed studies, Llanaj et al. [26] used food images and kitchen utensils commonly used in the target Roma population to estimate portion sizes. Ngo et al. [40] highlight the importance of tailoring portion size estimation methods to cultural contexts to mitigate misreporting influenced by cultural perceptions. Additionally, use of updated, region-specific food composition databases is essential for accurately assessing energy and nutrient intake. Among the studies analyzed in the review, two discussed the use of national food composition databases. However, the adequacy of existing national databases for reflecting the dietary habits and nutrient content of ethnic minority populations, like the Roma, remains a concern and could impact result reliability. Abu-Saad et al. [41] stress the importance of incorporating ethnic foods into food composition databases, as these ethnic foods can serve as key predictors of nutrient intake levels among ethnic minority populations. A food composition database for selected ethnic foods consumed in Europe was created by Khokhar et al. in 2010 [42]. Expanding this food composition data to include other ethnic minority groups, such as the Roma population, can facilitate the extraction of more reliable data on the composition of foods among ethnic minorities.

Ngo et al. highlighted the need for more research to address dietary knowledge gaps in marginalized groups, such as the Roma population, who face poverty, food insecurity, and low literacy [40]. Due to cultural specificities and low literacy, we recommend interview-based data collection instead of self-administered methods. For the Roma population, it’s advisable to use interview-based data collection methods conducted by well-trained interviewers who share the same ethnic or cultural background [43]. Llanaj et al. involved Roma university students in data collection, and Kozubik et al. emphasized letting participants express themselves freely. These culturally sensitive methods enhance understanding of the Roma’s dietary habits and nutrition challenges, improving the quality and validity of research findings.

To address the lack of validated instruments to assess ethnic minorities’ habitual diets Beukers et al. [44] developed ethnic-specific food frequency questionnaires that enable standardized and comparable assessment of the diet of five different ethnic groups. The authors of this review believe that there is a need to develop a specific food frequency questionnaire for the Roma population.

### Measuring dietary quality

Dietary quality indicators were employed to assess dietary quality in four studies in this review. Dietary quality scores are employed to classify diets based on their nutritional composition and/or quality and quantity of foods, aiding in the identification of healthy diets that can enhance overall dietary quality [45]. DQS offers valuable insights into dietary diversity, indicating food quality, and security. It helps pinpoint vulnerable individuals in the Roma population from socio-economic and nutritional standpoints. They allow for comparisons across populations or demographic groups to identify disparities in dietary patterns and their health implications. Current methodologies should be tested and adapted for diverse groups like the Roma, particularly in areas with limited food access.

### Qualitative methodology

Qualitative methods enable a high degree of interpretation and transformation of data within a well-defined framework facilitating a broader contextual understanding of specific social phenomena. Accordingly, studies conducted using these qualitative methods are particularly important within the Roma community. One study by Kozubik et al. [30] employed ethnography to highlight that the consumption of certain foods serves as a status symbol among the Roma, and their research also debunked several myths related to Roma nutrition. Another qualitative study, utilizing the Grounded Theory approach, shed light on the significance of attitudes towards overweight and obesity and culturally influenced behavior patterns as crucial determinants of eating habits [32].

The results of Kozubik et al. [30] raise the question if dietary acculturation and diet-related disparities exist among the Roma population. The findings from the Slovak study indicated that traditional Roma foods were also the traditional foods of poor Slovak peasants [30]. Furthermore, their results revealed that the dietary habits of those living in impoverished communities in eastern Slovakia still resemble to the people who had lived there two centuries ago, and there is no significant difference between the diets of Roma and non-Roma Slovaks. In contrast, several studies have identified significant differences in the dietary habits between Roma and the non-Roma majority population, attributing them to the existence of diet-related disparities. Diet-related disparities involve variations in diet, disease prevalence, mortality, and disease burden within specific population subgroups. It’s important to recognize that disparities are primarily shaped by socioeconomic status rather than solely racial or ethnic identity [46]. Contributing factors to these disparities are complex and include individual, social, and cultural characteristics, among others. Yau et al. [47] noted differences in diet quality among ethnic groups, finding that low socioeconomic status did not uniformly correlate with poor diet quality across all ethnicities. This discrepancy may stem from the preservation of traditional diets specific to certain ethnic groups, regardless of socioeconomic status.

To develop culturally appropriate dietary interventions and nutrition education programs, understanding the factors underlying diet behaviors in ethnic minority groups is a first step. Based on our findings, to assist in collecting dietary data among the Roma population, subjective dietary assessment methods combined with a qualitative, participatory approach may be suitable for this hard-to-reach group. The results obtained using qualitative methods enrich our knowledge about the dietary habits of the Roma and supplement the results of quantitative dietary assessment methods.

### The applicability of innovative dietary assessment technologies in the Roma population

The choice of dietary assessment method should align with the main research question, as demonstrated in the analyzed studies. For the quantitative determination of nutrient intake values, 24-h dietary recall was used, while studies aiming to gather general information about foods and food groups consumed used methods like FFQs or brief dietary assessment instruments, such as the consumption frequency of sugar-sweetened beverage consumption or fast-food consumption.

Some authors, such as Ngo et al. [40] highlighted the need for additional research to fill dietary knowledge gaps among certain populations more than a decade ago. They emphasized the importance of marginalized and hard-to-reach groups like the Roma population that face poverty and food insecurity, have a unique socio-demographic structure, and exhibit low levels of education that significantly affect their literacy skills. This raises the question of how to apply innovative dietary assessment technologies, such as personal digital assistants, smartphones, mobile applications, and sensor-based recording in low-resource settings and among populations with low literacy levels. Existing studies suggest that these innovative technologies are mainly used in industrialized countries and controlled environments. Their application is limited in low-resource settings because it requires high literacy, computer skills, internet access, imposes a high respondent and researcher burden, demands significant technical development and data processing efforts, and assumes that participants are familiar with new technologies and require training on their part as well [48]. If such methods are to be used in low-resource environments, their effectiveness must be evaluated. Furthermore, before the implementation of new technologies, validation studies should be conducted, especially within the Roma minority population.

We summarized the strengths, limitations, and applicability of the dietary assessment methods that could be used in the Roma population, based on the studies included in the review, synthesis of the guide on dietary assessment in the low-resource setting of FAO [49] and the authors’ own viewpoints (Table 3).

**Table 3:** Review of strengths, limitations, and practical feasibility of dietary assessment approaches in the Roma population.

Dietary assessment method	Strengths	Limitations	Feasibility in the Roma population
24-h dietary recall	Can assess the usual intakes of a large population, and can capture additional information such as preparation methods. The interview-based 24-h dietary recall does not require literacy.	Requires well-trained interviewers, recall bias may be present, multi-day recalls are needed to assess usual/general intake, and expensive.	The interview-based dietary recall is applicable with well-trained interviewers, and multi-day recall is needed.
Food frequency questionnaire	Low respondent burden, assesses usual intake over a long time period, can capture specific food group and portion size estimates, simple and cost-effective, an interview-based FFQ questionnaire does not require literacy.	Questionnaires require adaptation and validation to study the population, and do not give accurate information on the estimated portion size. The food list cannot be complete, risk of over- or underreporting.	The interview-based FFQ is applicable with well-trained interviewers, but ethnic foods need to be included in the food list, and other factors that could influence the diet of the study population need to be considered.
Brief dietary assessment	It can be used in a situation where a full-length FFQ questionnaire is not practical, can be used for surveillance, low participant burden, and is cost-effective and time-saving.	Does not give accurate data on intake, specific to research aims.	The interview-based assessment is applicable with well-trained interviewers in a situation where the full-length FFQ is not practical.
Tools for assessing dietary diversity	A good indicator for assessing dietary diversity, low participant burden, cost-effective, time-saving data collection and analysis.	Does not represent usual intake at the individual level. Qualitative dietary data collection is necessary for the (dietary diversity) analysis, assessment of the full picture of dietary quality of subjects is not possible.	Its application is useful for assessing dietary diversity, but the unit of analysis (household or individual) needs to be considered. Data interpretation needs to be cautious when indicators are validated through secondary data analysis.
Qualitative (in-depth interview)	Provides detailed information on eating habits, does not require literacy, and enables a deeper understanding of eating habits, traditions, and cultural differences.	Trained interviewers are required, time-consuming, high costs, specific to research aims and target population.	Its application is useful with well-trained interviewers after gradually developing trust with the participants.

## Limitations and strengths

The present review has several important limitations regarding its findings. In this review, we only included English-language publications, and due to language constraints, it was not possible to review studies in languages other than English.

Another limitation of the review is that the studies examining the dietary habits of Roma were conducted mostly in Central and Eastern European countries. Therefore, the results should be interpreted within this cultural, social, and economic context, thus not a global picture of the dietary habits of the Roma population was described. In Western European countries such as England or France, there is a significant Roma population and research on the health of the Roma is extensive. However, Aspinall [50] emphasizes that there are no comprehensive studies on nutritional issues in the Roma community, only anecdotal evidence is available. This underscores the persistent knowledge gap in dietary research among Roma people and the need for robust methodologies in future studies.

According to the review’s findings, the majority of the applied dietary assessment methods were not validated, which limits generalizability and reliability. Despite the limitations, it is important to emphasize that studies conducted in different countries drew similar conclusions regarding the dietary intake and eating habits of the Roma population.

## Conclusion

The results of this study revealed that the nutritional intake and food consumption of the examined Roma populations are inadequate, their adherence is low to selected dietary guidelines and food is a symbol of social status among them. The 24-h dietary recall, FFQ, and dietary habits questionnaire were commonly used to assess dietary outcomes in the Roma ethnic group. In the methodology descriptions of studies targeting the Roma community’s nutrition and dietary habits, several shortcomings were identified. The dietary assessment tools employed in most studies were not validated or adapted for the Roma community. Selecting the appropriate method and adapting the chosen methods to the Roma community is crucial for the reliability of the results. Furthermore, there is a need to develop specific dietary assessment methods for the Roma population. Only a small number of articles met the inclusion criteria of the review, highlighting a significant gap in the high-quality assessment of Roma nutrition that needs to be addressed. In addition to retrospective methods, it is worthwhile to use prospective direct and indirect dietary assessment methods in studying Roma nutrition. A comprehensive understanding of Roma nutrition and dietary culture is essential for planning effective nutrition-related interventions. Qualitative, participatory methods should be prioritized alongside quantitative approaches to explore dietary traditions, beliefs, and cultural differences, providing insights into minority-specific issues.

## Supplementary information

Table S1 PRISMA-ScR-Checklist

Table S2 Details of the search strategy used for collecting studies

Table S3 Information from studies that assessed nutrient intake by the 24-hour recall method

Table S4 Methodological details from studies that assessed diet by dietary quality scores and indices

Table S5 Methodological details from studies that assessed food consumption by FFQ

Table S6 Methodological information from studies used qualitative methods to assess nutrition

## Data Availability

All data generated or analyzed during this study are included in this published article and its supplementary information files.
