# Supplementary material for: Overview of dietary intake assessment methods and dietary outcomes in Roma population: a scoping review
Source: Eur J Clin Nutr. 2026 Jan 31;80(4):354–64. doi: 10.1038/s41430-025-01677-z (PMC13083244; doi:10.1038/s41430-025-01677-z)
Supplement: Supplementary file 2 — Table S2 Details of the search strategy used for collecting studies [file 41430_2025_1677_MOESM2_ESM.docx]

**Table S2** Details of the search strategy used for collecting studies

| **PubMed (**Title/Abstract**)**  ("Nutrition" OR "Food" OR "Meal" OR "Intake" OR "Diet*" AND ("Roma" OR "Romany" OR"Gypsy" OR "Gypsies" OR "Romani") NOT ("Gypsy moth") |
| --- |
| **Web of Science** (Topic)  ("Nutrition" OR "Food" OR "Meal" OR "Dish" OR "Intake" OR "Diet*" OR ”Eat*”) AND ("Gypsy" OR "Roma*" OR "Gypsies") NOT ("Gypsy moth") |
| **Scopus** (Title/Abstract/Keyword)  ("Nutrition" OR "Food" OR "Meal" OR "Dish" OR "Intake" OR "Diet*") AND ("Roma" OR "Romany" OR "Gypsy" OR "Gypsies") AND NOT ("Gypsy moth") |
