# Supplementary material for: Overview of dietary intake assessment methods and dietary outcomes in Roma population: a scoping review
Source: Eur J Clin Nutr. 2026 Jan 31;80(4):354–64. doi: 10.1038/s41430-025-01677-z (PMC13083244; doi:10.1038/s41430-025-01677-z)
Supplement: Supplementary file 3 — Table S3 Information from studies that assessed nutrient intake by the 24-hour recall method [file 41430_2025_1677_MOESM3_ESM.docx]

**Table S3** Information from studies that assessed nutrient intake by 24-hour recall method

| **Diet assessed by 24-hour recall method** | **Reference time** | **Recall number** | **Administration** | **Validation** | **Measurements of portion size** | **Food composition database** | **Analysis tool** | **Adaptation of study instruments** | **Result** |
| --- | --- | --- | --- | --- | --- | --- | --- | --- | --- |
| Llanaj et al. (2020) | 24 h | 2 | Interview | Yes | Food images and kitchen utensils | Database built in the nutricomp software | Nutricomp dietcad ver. 3.03 software | Images of canned foods commonly consumed, usual utensils and some containers widely used among subjects were included | Energy intake (kcal/day) among Hungarian Roma: 2214.1, Males 2212.5 (95% CI 2064.2–2360.8), Females: 2076.0 (95% CI 1994.5–2157.5 0.561). Energy intake among Hungarian non-Roma: 2188.3, Males 2270.9 (95% CI 2148.9–2392.8), Females 2113.1 (95% CI 2016.5–2209.7). Total carbohydrate daily intake, as energy percentage, was significantly higher among Hungarian Roma, but still significantly lower than the recommended range in both groups. Sugar intake was significantly higher than the recommended daily intake of 10% of total energy intake. Total dietary fiber intake was much lower than the recommended daily amount for both groups. Total dietary protein intake was significantly higher compared to recommended intake ranges among Hungarian non-Roma, but not among Hungarian Roma. |
| Sedova et al. (2018) | 24 h | 1 | Interview | Yes | NA |  | Nutridan II program |  | Daily energy intake was 1900.8 kcal in the Roma group, 2519.3 kcal in the control group. Index Protein % TEI: 16.7, Index Fat % TEI: 35.3 Index Saccharides % TEI: 44.8 in Roma population vs Index Protein % TEI: 17.0, Index Fat % TEI: 32.9 Index Saccharides % TEI: 44.9 in the control group. The average amount of dietary fiber was 19.5 in the Roma set and 20.2 in the control set. |
| Dolák et al. (2016) | 24 h | 1 | Interview | Yes | NA | NA | NA | NA | Dietary (energy, macro- or micronutrient) intake data was not reported. |
| Siváková et al. (2007) | NA | 1 | NA | NA | NA | Slovak food composition database | Self-developed software | NA | The mean intake of fat and protein was higher than the recommended dietary allowance with a high proportion of animal protein and cholesterol. The mean intake of vitamins was lower than the recommendation. |

References

1. Llanaj E, Vincze F, Kósa Z, Sándor J, Diószegi J, Ádány R. Dietary profile and nutritional status of the Roma population living in segregated colonies in Northeast Hungary. Nutrients. 2020;16;12(9):2836.
2. Sedova L, Tothova V, Novakova D, Olisarova V, Bartlova S, Dolak F, Kajanova A, Prokesova R, Adamkova V. Qualification of food intake by the Roma population in the Region of South Bohemia. International journal of environmental research and public health. 2018;15(2):386.
3. Dolák F, Sedova L, Nováková D, Olisarova V. Approach to prevention of obesity of Roma population in the Region of South Bohemia with focus on selected eating behaviors. Neuro Endocrinol. Lett. 2016;1;37:46-51.
4. Siváková D, Babinská K, Bašistová Z, Zacharová M, Wsólová L, Béderová A. Dietary patterns and lifestyle in a sample of a Slovak Romany community. Anthropologischer Anzeiger. 2007;1:25-35.
