# Supplementary material for: Overview of dietary intake assessment methods and dietary outcomes in Roma population: a scoping review
Source: Eur J Clin Nutr. 2026 Jan 31;80(4):354–64. doi: 10.1038/s41430-025-01677-z (PMC13083244; doi:10.1038/s41430-025-01677-z)
Supplement: Supplementary file 4 — Table S4 Methodological details from studies that assessed diet by dietary quality scores and indices [file 41430_2025_1677_MOESM4_ESM.docx]

**Table S4** Methodological details from studies that assessed diet by dietary quality scores and indices

| **Diet quality assessed by dietary quality scores** | **Data obtained for the analysis** | **Approach** | **Dietary Quality Scores** | **Level of food consumption information** | **Result** |
| --- | --- | --- | --- | --- | --- |
| Bárdos et al., 2022 | Nutrient data and individual food intake data | Health science | Healthy Eating Index-2015 | Individual | Overall low median HEI-2015 scores for the Roma and majority population groups, with significantly lower total score among Roma participants, compared to non-Roma. Intakes of fruits, greens and beans, whole grains, and plant proteins were low in both groups and significantly lower among Roma. Daily sodium intakes were more than double the recommendation. Roma’s diet contained significantly more added sugar. |
| Porras et al., 2022 | Food intake data | Health science | They developed indexes based on the consumption criteria defined in the Nutrition, Physical Activity, and Obesity Prevention (NAOS) strategy of the Ministry of Health | Individual | The differentiated analysis of various NAOS criteria showed a tendency of lower adherence by the Roma population to the guidelines recommended by the Ministry of Health. No differences were observed regarding occasional consumption of sweets or sugary drinks, nor in adherence to recommended breakfast patterns. |
| Llanaj et al., 2021 | Nutrient-based dietary patterns | Health science | Healthy Diet Indicator, Dietary Inflammatory Index, Dietary Approaches to Stop Hypertension, EAT-Lancet | Individual | Poor adherence to selected dietary guidelines in both populations. Roma ethnicity was associated with a lower Dietary Inflammatory Index. |
| Ciaian et al., 2018 | Household budget survey data | Econometric | Diversity measured by Simpson index and by Entropy index | Household | There was a significant difference in diet diversity between Roma and non-Roma ethnic groups: Roma’s diet diversity is lower by 15-18%. The average Roma’s diet contains a higher proportion of cereals and a lower proportion of dairy products and fruits and vegetables. |

References

1. Bárdos H, Llanaj E, Vincze F, Diószegi J, Pikó P, Kósa Z, Sándor J, Ádány R. Diet quality as assessed by Healthy Eating Index-2015 among Hungarian Roma living in settlements of Northeast Hungary. Scientific Reports. 2022;10;12(1):19213.
2. Arza Porras J, Rodríguez Camacho MF, Carrón Sánchez J. Dietary patterns of Roma population and total population in Spain. 2022. (No. ART-2022-127428).
3. Llanaj E, Vincze F, Kósa Z, Bárdos H, Diószegi J, Sándor J, Ádány R. Deteriorated dietary patterns with regards to health and environmental sustainability among Hungarian Roma are not differentiated from those of the general population. Nutrients. 2021;13(3):721.
4. Ciaian P, Cupák A, Pokrivčák J, Rizov M. Food consumption and diet quality choices of Roma in Romania: A counterfactual analysis. Food security. 2018;10:437-56.
