# Supplementary material for: Overview of dietary intake assessment methods and dietary outcomes in Roma population: a scoping review
Source: Eur J Clin Nutr. 2026 Jan 31;80(4):354–64. doi: 10.1038/s41430-025-01677-z (PMC13083244; doi:10.1038/s41430-025-01677-z)
Supplement: Supplementary file 5 — Table S5 Methodological details from studies that assessed food consumption by FFQ [file 41430_2025_1677_MOESM5_ESM.docx]

**Table S5** Methodological details from studies that assessed food consumption by FFQ

| **Diet assessed by food frequency questionnaire** | **Reference time** | **Administration** | **Validation** | **Number of foods/food categories** | **Inclusion of ethnic foods in the FFQ** | **Source** | **Adaptation of study instruments** | **Result** |
| --- | --- | --- | --- | --- | --- | --- | --- | --- |
| Olišarová et al. (2018) | Weekly | Interview | NA | 13 | NA | NA | NA | A higher consumption was reported of high-sugar drinks and foods (3–4 Times a week) compared to fruits and vegetables (not at all or only 1–2 times a week). Chicken, pork and beef was the most frequently consumed meat. Potatoes, pasta, rice and dumplings were most commonly eaten side dishes (1–2 times a week). |
| Sedova et al. (2018) | NA | Interview | NA | NA | NA | NA | NA | Fruits and vegetables consumption is lower in the Roma population than the control group. A small part of respondents from both groups consume fruits and vegetables several times a day. |
| Dolák et al. (2016) | NA | Interview | NA | NA | NA | NA | NA | Roma eats less regularly than the majority population. Roma ate significantly more often in fast food restaurants (3 to 4× per week) than the majority population). |
| Hijová et al. (2014) | Monthly | Interview | NA | 28 | NA | NA | NA | Consumption of fruit, vegetables and dairy products were significantly lower in Roma than non-Roma respondents. Roma females consumed significantly more meat and soft drinks. No differences were found between Roma and non-Roma in the consumption of meat products and farinaceous dishes. |
| Hoxha et al. (2013) | NA | Interview | NA | The most common food items | NA | NA | NA | Two dietary patterns were identified: one with a higher consumption of dairy products, vegetables, and milk and another pattern included fat, bread and alcohol. |
| Rambousková et al. (2009) | Monthly | Interview | NA | Food groups that are sources of fat, sugar, vitamins, and trace elements | NA | NA | NA | Roma women exhibited a higher prevalence of consuming white bread, yeast rolls, pâtés, liver, smoked sausages, French-fried potatoes, potato chips, crisps, deli products, and sweet beverages. In contrast, non-Roma women reported more frequent consumption of fish, cheese, fruit, fruit juices, vegetables, bread, and whole-meal products, and they favored margarine over butter or lard. No statistically significant differences were observed in the consumption of meat, poultry, milk, yogurt, and eggs between the two groups. |
| Siváková et al. (2007) | NA | NA | Yes | NA | NA | NA | NA | Food consumption was not reported, the result of dietary intake is summarized in Table S3. |

References

1. Olišarová V, Tóthová V, Bártlová S, Dolák F, Kajanová A, Nováková D, Prokešová R, Šedová L. Cultural features influencing eating, overweight, and obesity in the Roma people of South Bohemia. Nutrients. 2018;28;10(7):838.
2. Sedova L, Tothova V, Novakova D, Olisarova V, Bartlova S, Dolak F, Kajanova A, Prokesova R, Adamkova V. Qualification of food intake by the Roma population in the Region of South Bohemia. International journal of environmental research and public health. 2018;15(2):386.
3. Dolák F, Sedova L, Nováková D, Olisarova V. Approach to prevention of obesity of Roma population in the Region of South Bohemia with focus on selected eating behaviors. Neuro Endocrinol. Lett. 2016;1;37:46-51.
4. Hijová E, Gecková AM, Babinská I, HepaMeta Team. Do eating habits of the population living in Roma settlements differ from those of the majority population in Slovakia. Cent Eur J Public Health. 2014;1;22(88):S65-8.
5. Hoxha A, Dervishi G, Bici E, Naum A, Seferi J, Risilia K, Tresa E. Assessment of nutritional status and dietary patterns of the adult Roma community in Albania. AMJ. 2013;3:32-8.
6. Rambousková J, Dlouhý P, Křížová E, Procházka B, Hrnčířová D, Anděl M. Health behaviors, nutritional status, and anthropometric parameters of Roma and non-Roma mothers and their infants in the Czech Republic. J Nutr Educ Behav. 2009;41(1):58-64.
7. Siváková D, Babinská K, Bašistová Z, Zacharová M, Wsólová L, Béderová A. Dietary patterns and lifestyle in a sample of a Slovak Romany community. Anthropologischer Anzeiger. 2007;1:25-35.
