# Supplementary material for: Overview of dietary intake assessment methods and dietary outcomes in Roma population: a scoping review
Source: Eur J Clin Nutr. 2026 Jan 31;80(4):354–64. doi: 10.1038/s41430-025-01677-z (PMC13083244; doi:10.1038/s41430-025-01677-z)
Supplement: Supplementary file 6 — Table S6 Methodological information from studies used qualitative methods to assess nutrition [file 41430_2025_1677_MOESM6_ESM.docx]

**Table S6** Methodological information from studies used qualitative methods to assess nutrition

| **Diet assessed by qualitative methods (semi-structured and in-depth interviews)** | **Administration** | **Question type** | **Source of questions** | **Qualitative method** | **Theory** | **Analysis tool** | **Adaptation to study population** | **Main topic identified** | **Result** |
| --- | --- | --- | --- | --- | --- | --- | --- | --- | --- |
| Olišarová et al. (2018) | Face-to-face interview | Open-ended | NA | Grounded theory | NA | MAXQDA 11 | NA | -food intake  -motivation for foodstuff selection -preferred preparation of foodstuff -place of eating -number of meals a day -frequency of eating | The Roma may be at increased risk of overweight and obesity due to irregular eating and a culture of moderate overconsumption. Eating is a measure of the family status. Roma families cook a lot, particularly after the payment of wages of social or other benefits. |
| Kozubik et al. (2018) | Face-to-face interview | Open-ended | NA | Ethnography | Classical sociological theory | NA | NA | -food is a measure of wealth,  -food storage,  -traditional Roma foods, eating habits | Eating serves as a symbol of social status in the Roma community. Typical Roma foods were the traditional foods of Slovak residents living in poverty. Food and eating habits of the Roma have not greatly changed over the centuries. |

References

1. Olišarová V, Tóthová V, Bártlová S, Dolák F, Kajanová A, Nováková D, Prokešová R, Šedová L. Cultural features influencing eating, overweight, and obesity in the Roma people of South Bohemia. Nutrients. 2018;28;10(7):838.
2. Kozubik M, Van Dijk JP, Odraskova B. Roma housing and eating in 1775 and 2013: a comparison. International journal of environmental research and public health. 2018;15(4):588.
